# The Gut Entomotype of Red Palm Weevil *Rhynchophorus ferrugineus* Olivier (Coleoptera: Dryophthoridae) and Their Effect on Host Nutrition Metabolism

**DOI:** 10.3389/fmicb.2017.02291

**Published:** 2017-11-21

**Authors:** Abrar Muhammad, Ya Fang, Youming Hou, Zhanghong Shi

**Affiliations:** ^1^State Key Laboratory of Ecological Pest Control for Fujian and Taiwan Crops, Fujian Agriculture and Forestry University, Fujian, China; ^2^Fujian Provincial Key Laboratory of Insect Ecology, College of Plant Protection, Fujian Agriculture and Forestry University, Fujian, China

**Keywords:** symbiotic invasion, gut microbiota, *Rhynchophorus ferrugineus*, insect symbiosis, cellulose degradation

## Abstract

For invasive insects, the potential roles of gut microbiota in exploiting new food resources and spreading remain elusive. Red palm weevil (RPW), *Rhynchophorus ferrugineus* Olivier, is an invasive destructive pest which feeds on nutrient-poor tender tissues and has caused extensive mortality of palm trees. The microbes associated with insects can improve their nutrition assimilation. However, experimental evidence on the interactions between RPW and its gut microbiota is still absent. The aim of this study is to determine the dynamics changes and the bacterial entomotype in the RPW gut and its potential physiological roles. Here, we confirmed RPW harbors a complex gut microbiota mainly constituted by bacteria in the families *Enterobacteriaceae, Lactobacillaceae, Entomoplasmataceae*, and *Streptococcaceae*. RPW gut microbiota exhibited a highly stable microbial community with low variance in abundance across different life stages and host plants. Furthermore, the abundance of *Enterobacteriaceae* was markedly increased but that of *Acetobacteraceae* was reduced significantly after administration of antibiotics. Although no significant effects were found on the body weight gain of RPW larvae, these alterations dramatically decreased the concentration of hemolymph protein and glucose while that of hemolymph triglyceride increased. In the gut of wild-caught RPW larvae, seven bacterial species in the genera *Klebsiella, Serratia, Enterobacter*, and *Citrobacter* were shown to have an ability to degrade cellulose. Together, RPW accommodate a stable gut microbiota which can degrade plant polysaccharides and confer their host optimal adaptation to its environment by modulating its metabolism.

## Introduction

*Rhynchophorus ferrugineus*, also known as the red palm weevil (RPW), is widely considered as the most devastating insect pest of palms in the world, even in the countries where it has been accidently introduced (Ju et al., 2011; Hou et al., 2011; Wan et al., 2015). This weevil is native to southern Asia and Melanesia, but due to international exchange of infected plant materials, it has spread to the Middle East, Africa and the Mediterranean Basin. More recently, RPW has become established in China, Japan, Australia and the Caribbean (Zhang et al., 2003; Wang et al., 2015). In China, almost 20,000 coconut trees have been killed, with the area of damage by RPW covering over 10,000 km^2^ since its invasion, and it has seriously threatened the green ecological security of China’s coastal areas (Li et al., 2009; Ju et al., 2011; Pu and Hou, 2016; Ge et al., 2016).

The development of RPW from egg to newborn adult occurs in the palm tree trunk and the weevil larvae feed on tender tissues and sap within the apical growing point of palms (Butera et al., 2012; Shi et al., 2014). The resulting damage is often only visible long after infestation when palms are close to death (Faleiro, 2006). Both palm tissues and sap are rich in carbohydrates such as cellulose, hemicelluloses, lignin, sucrose and glucose, but poor in assimilable nitrogen sources (Nagnan et al., 1992; Behar et al., 2005; Morales-Jiménez et al., 2009). For this reason, they represent a non-easily digestible and nutrient-poor substrate for most eukaryotes.

As the most diverse biological group, insects have been found in almost every possible nutritional niche on Earth, and with a remarkable adaptation to a wide range of diets including dead wood, feathers or blood, seem incapable of sustaining basic nutritional demands. More and more evidence indicates that this capacity is not from their own unique metabolic flexibility but is conferred by their microbiota (Douglas, 2009, 2015; Storelli et al., 2011; Engel and Moran, 2013; Ben-Yosef et al., 2014; Sugio et al., 2016; Xu et al., 2016). For bark- and wood-boring species, symbioses with cellulose-degrading microorganisms appear to compensate for the inability to synthesize cellulases in many insects (Martin, 1991; Douglas, 2009, 2015; Adams et al., 2011), and these obligate or facultative associations are important factors driving the evolution of wood-feeding insects (Veivers et al., 1983). For instance, the prominent invasive pests, such as the *Dendroctonus* bark beetles, wood-boring emerald ash borer and tree-killing wasp, *Sirex noctilio*, are inhabited by gut microorganisms that contribute to host nutrition by degradation of cellulose (Vasanthakumar et al., 2008; Butera et al., 2012) and hemicelluloses (Adams et al., 2011; Butera et al., 2012) and nitrogen fixation (Morales-Jiménez et al., 2013).

Invasive insects, such as *Brontispa longissima* and *Dendroctonus valens*, have caused huge losses to economic and ecosystem services and human health (Lu et al., 2016; Wan and Yang, 2016). Unfortunately, it is less widely appreciated that these insect species often derive increased opportunities for establishment and cause additional environmental and economic loss because of their associations with symbiotic microorganisms. There are three potential threats arising from symbiotic microorganisms of invasive insects. First, symbionts may contribute to the damage caused by their insect host. The prominent example is whitefly *Bemisia tabaci* and its vectored begomoviruses such as tobacco curly shoot virus and tomato yellow leaf curl China virus which extensively damaged tomato and tobacco crops in southern China (Jiu et al., 2007). Second, symbionts may be released from their host and thus begin a subsequent invasion (Mota et al., 2006; Zhao et al., 2013, 2014). Finally, and probably least understood, symbionts play important roles in the survival and reproduction of their host insects during establishment in new regions (Zhou et al., 2015; Lu et al., 2016). Therefore, elucidation of the complex interactions between invasive insects and their associated microbes not only provide important information towards revealing the mechanisms underpinning their invasion success, but also drive the development of novel and more effective management approaches.

Considering the huge economic and environmental damages caused by RPW, the interest in this pest has markedly increased in recent decades. Most studies paid attention to the efficacy of different chemical and bio-control strategies (Llácer et al., 2012a,b; Mazza et al., 2014; Peng et al., 2016; Pu et al., 2017). Recently, some preliminary investigations have revealed the gut bacterial community associated with RPW larvae (Khiyami and Alyamani, 2008; Butera et al., 2012; Valzano et al., 2012; Jia et al., 2013; Tagliavia et al., 2014) and adults (Montagna et al., 2015), with some of these studies being done in the context of identifying potential insect pathogens for use in biological control (Butera et al., 2012; Valzano et al., 2012). Bacterial species with the ability to degrade polysaccharides and sucrose by hydrolase activity (e.g., *Klebsiella pneumonia* and *Lactococcus lactis*) have been identified in the gut of RPW larvae (Khiyami and Alyamani, 2008; Jia et al., 2013). Current reports on the gut microbiota of RPW have only focused on its larval and adult stage. For holometabolous insects, factors such as environmental conditions, life stages and diets have strong effects on shaping the structure and function of gut microbiota (Dethlefsen et al., 2007; Lehman et al., 2009; Sharon et al., 2010; Wang et al., 2011; Sudakaran et al., 2012; Montagna et al., 2015). Therefore, a comprehensive characterization of gut microbial dynamics across developmental stages of RPW to uncover the existence of entomotype which refers to the bacterial taxa consistently associated with the host (Montagna et al., 2016), is the prerequisite for understanding how a microbial population functions in the gut ecosystem. Unfortunately, the relationships between microbial dynamics and RPW health remain poorly understood. Given that symbionts are involved in driving the diversification and success of insects, we posit that bacteria harbored in the gut of RPW are playing pivotal roles in the host nutrition metabolism during invasion. In this study, comparisons on gut microbiota of RPW across different life stages and larvae from different host plants were performed to determine the dynamics changes and the entomotype in the RPW gut. Furthermore, the potential effects of gut microbiota on the nutrition metabolism of RPW are detected by the administration of antibiotics to disturb the inherent homeostasis of RPW gut microbiota.

## Materials and Methods

### Insect Sampling and Rearing

To detect the effect of developmental stages on gut microbiota, RPW larvae, pupae and adults were collected from *Cocos nucifera* L. in Wenchang City, Hainan Province in May 2014. Larvae from *Phoenix canariensis* were collected in Longyan City, Fujian Province in August 2014. The live RPW individuals were saved in plastic boxes with some soft palm tissues and then transported to Fujian Province Key Laboratory of Insect Ecology, Fujian Agriculture and Forestry University (FAFU). A laboratory population of RPW was established by adults that were trapped in Jinshan campus of FAFU (Supplementary Table S1). In our laboratory, RPW larvae were reared on sugarcane stem in the climatic chamber at 27 ± 1°C, 75% relative humidity (RH) and a photoperiod of 24 h dark, while adults were maintained under the following conditions: 27 ± 1°C, 75% RH, 12 h light/12 h dark. RPW larvae from *C*. *nucifera, P*. *canariensis* and our laboratory were employed to determine the influence of host plant species on its gut microbiota. All the wild-caught specimens were processed immediately in the laboratory for gut dissection and the extraction of total gut bacteria DNA upon arrival.

### Gut Dissection and DNA Isolation

Before dissection, all specimens (Seventh instar larvae, pupae, and adults) were surface-sterilized in 75% ethanol for 90 s, followed by three rinses in sterilized distilled water. From RPW adults, elytra and membranous wings were removed before sterilization. Under a stereomicroscope, gut dissections were conducted in a clean Petri dish (90 mm in diameter) with sterile phosphate-buffered saline (PBS) using sterilized scissors and forceps. Three guts were pooled in 1 ml sterile PBS as a replication and each treatment had three replications.

For pyrosequencing, DNA was extracted using a DNeasy Blood & Tissue Kit (Qiagen) following a protocol modified from the manufacturer’s guidelines for Gram-positive bacteria. Briefly, samples were hand-homogenized in 20 mM Tris-HCl (pH 8.0), 1.2% Triton X-100, 2 mM sodium EDTA containing 20 mg/ml lysozyme. Gut homogenates were centrifuged for 10 min at 5000 *g* under room temperature and the bacterial pellets were re-suspended in 180 μl enzymatic lysis buffer. The final elution step was repeated twice with 100 μl of AE buffer for greater yield. The quality of DNA samples was checked by electrophoresis on 1% agarose gel, and quantified using Nanodrop 1000 (Thermo Scientific).

### Illumina High-Throughput Sequencing of 16S rRNA Gene Sequences

The variable region 4 (V4) of the bacterial 16S rRNA gene was amplified with the general 16S rRNA primers 515F (5′-GTGYCAGCMGCCGCGGTA-3′) and 806R (5′-GGACTACHVGGGTWTCTAAT-3′). The PCR reactions were carried out in a total volume of 30 μl which comprised 15 μl Phusion^®^ High-Fidelity PCR Master Mix (New England Biolabs), 0.2 μM forward and reverse primers and 10 ng template DNA. The thermal conditions set for PCR were as follows: initial denaturation for 1 min at 98°C followed by 30 cycles of denaturation for 10 s at 98°C, annealing for 30 s at 50°C and elongation for 60 s at 72°C, then a final extension for 5 min at 72°C. The amplified PCR products were mixed with the same volume of 1× buffer containing SYBR green and run on a 2% agarose gel. Next, DNA from an aliquot of each PCR reaction was purified using a Qiagen Gel Extraction Kit and equal volumes of the three reaction products per sample were mixed together and the sequencing libraries were generated with TrueSeq^®^ DNA PCR-Free Sample Preparation Kit (Illumina). The libraries were sequenced using MiSeq platform after quality assessment on the Qubit@ 2.0 fluorometer (Thermo Scientific) and Agilent Bio-analyzer 2100 system.

The paired-end sequencing reads were merged through FLASH^1^ (V1.2.7) (Magoč and Salzberg, 2011). The raw tags were strictly filtered (<30 Phred score) to obtain high-quality clean tags using Quantitative Insight into Microbial Ecology (QIIME) software^2^ (V1.7.0) (Caporaso et al., 2010; Bokulich et al., 2013) and were aligned with Gold database by running the UCHIME algorithm to remove the chimera for effective tags (Edgar et al., 2011).

Operational taxonomic units (OTUs) were clustered using Uprase^3^ (V7.0.1001) to group the effective tags into OTUs with ≥97% sequence identity (ID) (Edgar, 2013). The most abundant unique sequence of each OTU cluster was selected as representative, and taxonomy of the non-chimaeric sequences was assigned by RDP Classifier^4^ (version 2.2) (Wang et al., 2007) using GreenGene database^5^ with default settings (DeSantis et al., 2006). To investigate the species richness and community diversity of samples or groups, six diversity indices, Chao 1, ACE, Shannon, and Simpson’s were calculated with the software package QIIME, version 1.7.0. Beta diversity analysis of RPW gut bacterial communities was carried out through PCoA (Principal Coordinate Analysis) and PCA (Principal Component Analysis) using QIIME (Version 1.7.0). ANOSIM (Analysis of similarity) was used to estimate the differences between gut bacterial communities associated with RPW at different life stages and larvae from different host plants.

### Bacterial Isolation and Identification

The larvae of *R. ferrugineus* were collected from the infested coconut palms *C*. *nucifer* in Hainan, China in mid-May 2014. The specimens were transported in plastic boxes along with host plant tissue to our laboratory. Upon arrival, larvae guts were dissected as above and homogenized by hand. The gut homogenates were poured on nutrient agar (NA) media in triplicate after serial dilution (10^-1^ to 10^-6^), and were incubated aerobically at 30°C for 24 h. Based on their color, size, and morphology, 200 single colonies were picked and repeatedly streaked on NA media to obtain pure cultures (Indiragandhi et al., 2007). The purified single colonies were inoculated in nutrient broth (NB) and incubated at 37°C for 24 h. Thereafter, 500 μl bacterial solutions of each colony were processed for DNA extraction with the TIANamp Stool DNA Kit DP 328 (TIANGEN) according to the manufacturer’s instructions. The bacterial species were identified as described by Butera et al. (2012) with the universal bacterial primers 27F (5′-AGAGTTTGATCATGGCTCAG-3′) and 1492 R (5′-TACGGYTACCTTGTTACGACTT-3′). The taxonomy of each 16S rRNA gene sequence was assigned with NCBI BLAST^6^ and Sequence Match in the Ribosomal DNA Project^7^ (RDP). Our representative bacterial sequences and those retrieved from the GeneBank database were aligned with ClustalW using MEGA 5.05 software.

To determine the ability of bacterial isolates to degrade cellulose, each of them was streaked on CMC (carboxymethyl-cellulose, Sigma) agar media and the plates were aerobically incubated at 37°C for 14 days. After growth, the incubated plates were covered with Congo red dye solution (Huang et al., 2012). After dye removal, cellulose degradation was indicated by the clear zone around the colonies. The cellulolytic index was calculated based on the ratio of the diameter of the clear zone to the diameter of the bacterial colony.

### Effect of Gut Microbiota on the Nutrition Metabolism of RPW

To evaluate the role of gut microbiota on host nutrition metabolism, an antibiotic cocktail containing kanamycin, tetracycline, gentamicin, and erythromycin (Chu et al., 2013) with the final concentration of 600 mg/L was used to disrupt the inherent structure of RPW gut microbiota. The fourth-instar larvae from the laboratory population were randomly allocated to the following two groups: control group (CK) and antibiotic group (AT). Before treatment, the initial body weight of each individual was measured with an electric microbalance (METTLER TOLEDO AL104). The treatments were conducted for a duration of 28 days. At the end of treatment, some larvae were dissected to extract the total DNA of gut microbiota for pyrosequencing to determine the antibiotic feeding-mediated changes in the community structure of the gut microbiota. The pyrosequencing and data analysis were completed as above.

The remaining larvae were used to extract the hemolymph for the analysis of metabolic indices, containing glucose, protein, and triglyceride (TAG) concentration. Before hemolymph collection, larvae were washed under running water to remove excrements and food particles, and were then anesthetized for 3–5 min on ice to immobilize them. The larvae epidermis was pierced by a fine sharp needle and 5 μl hemolymph of each larva was collected into labeled 1.5 ml clean microcentrifuge tubes, immersed into an ice box, and containing 2 μl of 0.2% phenylthiourea (PTU) to inhibit the melanization of hemolymph. Hemolymph collections were carried out at four time points of 7, 14, 21, and 28 days after feeding. Glucose, protein, and TAG concentrations were measured with a Glucose Measurement Kit (Shanghai Rongsheng Biological Pharmaceutical Co.), a BCA Protein Assay Kit (Tiangen PA115, Tiangen Biotech) and a Triglyceride Assay Kit (Zhejiang Dongou Diagnostic Products), respectively. These assays were done with a spectrometric reader (Molecular Device^®^) under the manufacturer’s protocol. Ten larvae were treated in each replication and each treatment comprised three replications.

### Statistical Analysis

Differences in the abundance of gut bacteria across developmental stages and host species at family and genus level, the cellulolytic ability of isolated bacterial species and that of nutrition indices were assessed by analysis of variance (ANOVA). The differences in abundance of gut bacteria and body weight between CK and AT groups were statistically detected by independent *t*-test. All analyses were performed using IBM SPSS Statistics (22.0). The significance level to threshold was set at 0.05 (*P* < 0.05).

## Results

### Ontogenetic Changes of RPW Gut Microbiota

A total of 733,855 raw reads from 10 RPW samples at different developmental stages (Seventh instar larvae, pupae, and adults) were characterized with the Illumina high-throughput sequencing. After quality trimming, 658,240 high-quality clean tags were obtained, which were binned into 5737 OTUs at the 3% distance level. RPW pupae had the highest species richness (Chao 1, ACE, and observed species) and the most diverse bacterial community being indicated by both Shannon and Simpson’s index, followed by larvae and adults, respectively (**Table 1**). The phyla *Proteobacteria* and *Firmicutes* accounted for the vast majority of reads (>93%) from all life stages. The third most abundant phylum in larvae and adults was *Tenericutes* at 5.0 and 1.8%, respectively. However, in pupae, *Actinobacteria* was the third dominant phylum at 1.11%. More than 90% of the reads were classified at the family level and 60 families were detected in the RPW gut at the abundance ≥0.01%, with *Enterobacteriaceae* representing 64.7% of the assemblage, followed by *Lactobacillaceae* (4.2%) and *Streptococcaceae* (1.97%). Twelve bacterial genera with the abundance ≥1% were detected and *Erwinia* (4.3%) is the most represented genus in the RPW gut, followed by *Lactococcus* (1.94%), *Entomoplasma* (1.66%), and *Erysipelothrix* (1.37%). Another 72 genera are represented at a value between 0.01 and 1%.

**Table 1:** Richness and diversity estimation of the gut bacterial communities associated with *Rhynchophorus ferrugineus* during its different life stages from the pyrosequencing analysis.

Sample	Number of reads	Number of OTUs	Community diversity		Species richness	
			Shannon	Simpson	Chao1	ACE
HL1	86,024	492	3.2	0.7	1052.8	929.5
HL2	99,929	311	3.6	0.8	460.8	545.2
HL3	78,582	294	2.8	0.7	413.1	440.0
HP1	33,544	597	3.9	0.8	896.9	946.4
HP2	81,496	697	4.2	0.8	1026.1	1095.9
HP3	66,927	621	3.8	0.7	691.9	705.0
HA1	34,900	245	2.5	0.7	379.4	403.0
HA2	57,071	283	2.5	0.7	423.6	468.1
HA3	57,715	273	1.7	0.4	366.4	408.6
HA4	62,052	445	2.1	0.4	623.4	736.5

**FIGURE 1:**
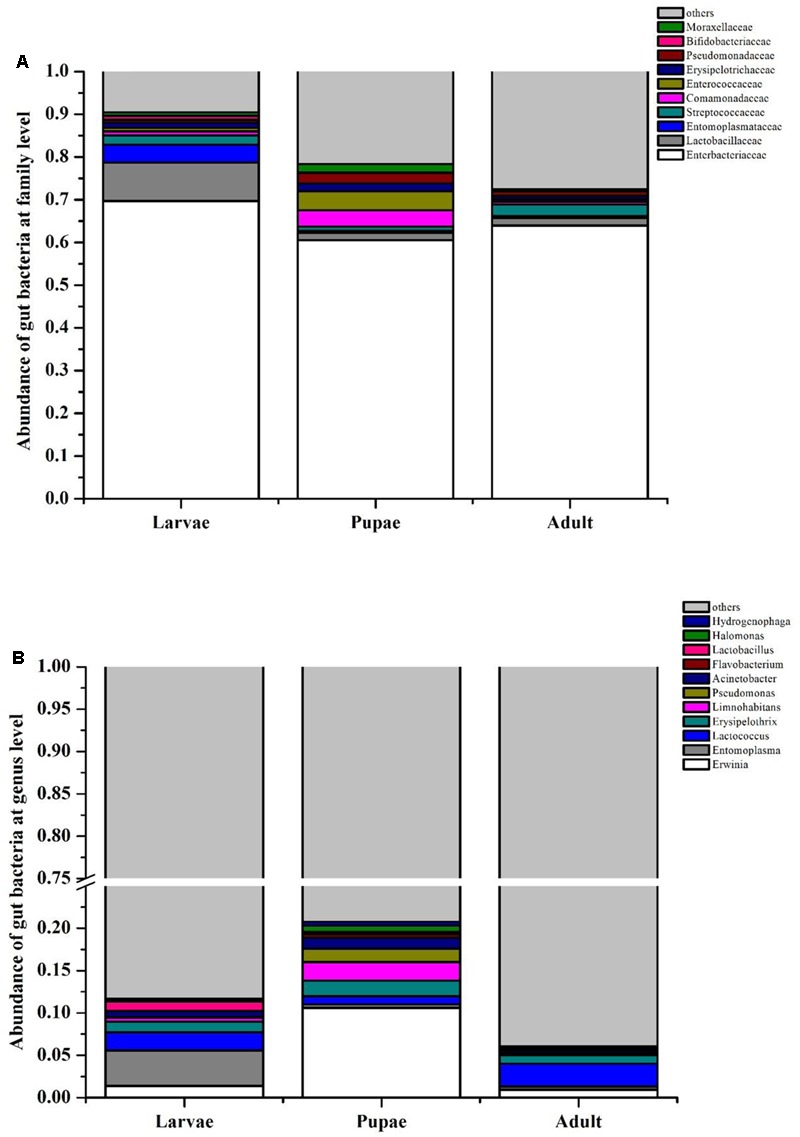
Bacterial community composition at family **(A)** and genus **(B)** level in the gut of *Rhynchophorus ferrugineus* during its different life stages.

In PCA analysis, the first two components PC1 and PC2 accounted for 51.62% (PC1 = 28.4%, PC2 = 23.22%) of the variation among the gut microbiota of RPW at different life stages (Supplementary Figure S1A). While in PCoA analysis, the PC1 and PC2 coordinates explained a total variation of 41.73% (PC1 = 27.26%, PC2 = 14.47%) (Supplementary Figure S1B). The ANOSIM analysis confirmed that there were no significant differences in the bacterial communities associated with RPW at different life stages (Adult-Larvae *p* = 0.4, Pupae–Larvae *p* = 0.2, Pupae–Adult *p* = 0.35). The family *Enterobacteriaceae* prominently dominated, representing ∼60% of the assemblage, in the larvae, pupae, and adult stages. With the transition from larvae to pupae, the percentage of *Lactobacillacea, Entomoplasmataceae*, and *Streptococcaceae* decreased to 1.76, 0.40, and 0.99%, respectively. However, *Enterocococcaceae, Comamonadaceae, Erysipelotrichaceae*, and *Moraxellaceae* became more rich to 4.44, 3.85, 1.85, and 1.96%, respectively (**Figure 1A**). In pupal stage, the relative abundance of genera *Erwinia, Acinetobacter, Pseudomonas*, and *Limnohabitans* increased to 10.60, 1.30, 1.56, and 2.21%, respectively, and *Erwinia* were strongly represented, but the percentage of *Entomoplasma, Lactococcus* and *Lactobacillus* dropped to 0.40, 0.96, 0.27%, respectively. During the transition from pupae to adult, only *Lactococcus* in the family *Streptococcaceae* became more abundant (**Figure 1B**). These results support that the gut bacterial compositions of RPW differed in abundance across the developmental stages. Classifier analysis revealed that the 375 OTUs, common to all three developmental stages, were comprised of 56 genera in the phyla *Proteobacteria, Firmicutes, Tenericutes* and *Actinobacteria*, with *Erwinia, Entomoplasma, Lactococcus, Lactobacillus, Pseudomonas, Limnohabitans*, and *Acinetobacter* appearing the most frequent genera, constituting the “entomotype” of RPW.

### The Effect of Host Plant Species on the Gut Microbiota of RPW Larvae

**FIGURE 2:**
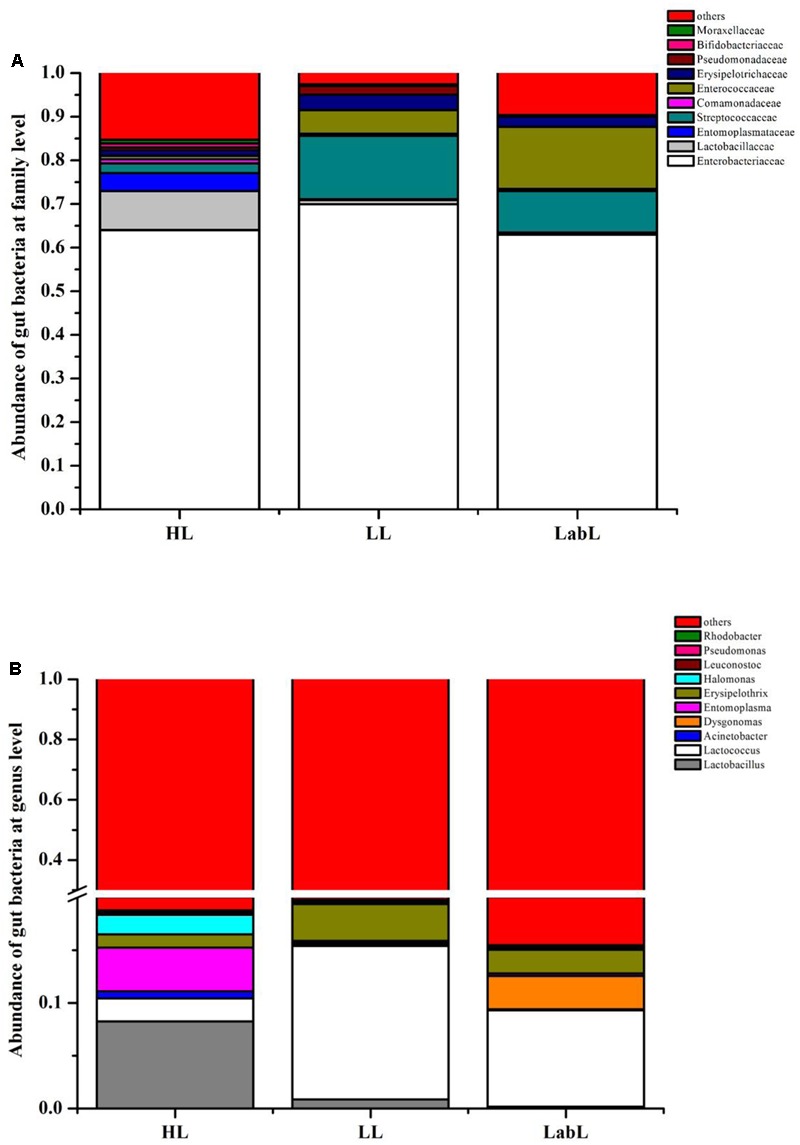
Relative abundance of gut bacterial families **(A)** and genera **(B)** in the larva of *Rhynchophorus ferrugineus* from different host plants. HL, LL, and LabL represent the Red palm weevil (RPW) larvae from *Cocos nucifera, Phoenix canariensis* and sugarcane, respectively.

Principal component analysis showed that the first two components PC1 and PC2 accounted for 41.63% (PC1 = 25.28%, PC2 = 16.35%) of the variation (Supplementary Figure S2A). Meanwhile, the PCoA analysis, PC1 and PC2 explained a total variation of 44.06% (PC1 = 28.78%, PC2 = 15.28%, Supplementary Figure S2B). RPW larvae from different host plant species bore the gut microbiota containing the same compositions just with variations in their abundance (**Figure 2**) and no marked differences were detected in the bacterial communities associated with all three larvae groups by an ANOSIM analysis (*P* > 0.05). RPW larvae from three species of host plants were inhabited with a similar bacterial community which was mainly composed of *Enterobacteriaceae, Lactobacillaceae, Entomoplasmataceae, Streptococcaceae, Enterocococcaceae, Erysipelotrichaceae*, and *Comamonadaceae*. The most dominant bacterial taxa was *Enterobacteriaceae*, accounting for over 60% of the reads (**Figure 2A**). At the genus level, gut microbiota of larvae from *C*. *nucifera* mainly comprised *Lactobacillus* (8.26%), *Entomoplasma* (4.14%), and *Lactococcus* (2.17%). In contrast, *Lactococcus* and *Erysipelothrix* represented 14.55 and 3.51%, respectively, in the gut of RPW larvae from *P*. *canariensis*. In the larvae from laboratory populations, *Lactococcus* (9.13%), *Dysgonomonas* (3.16%), and *Erysipelothrix* (2.26%) comprised the major gut bacterial taxa (**Figure 2B**). Together, comparisons on the gut bacterial community of RPW individuals across different developmental stages and host plants revealed that the gut microbiota of RPW exhibited a highly stable microbial community with low variance in compositions.

### Isolation and Cellulose Degradation Assay of Bacterial Isolates from RPW Larvae Gut

**FIGURE 3:**
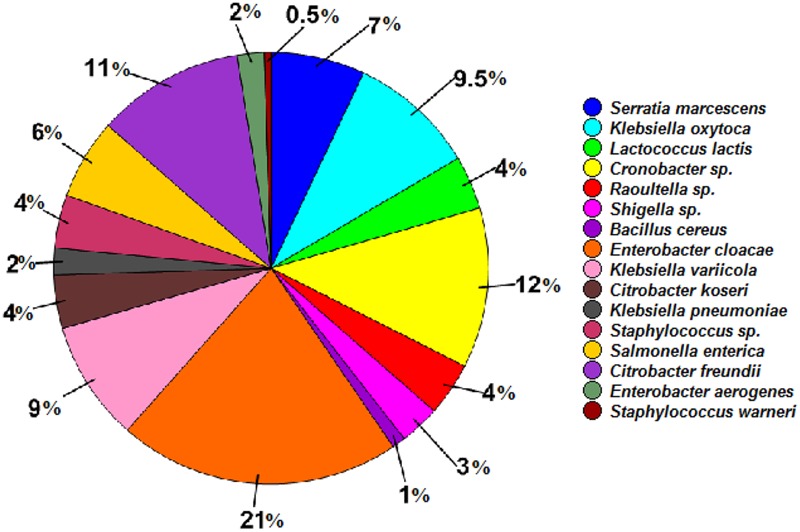
Relative abundance of cultivable bacterial species isolated from the wild-caught larvae of *Rhynchophorus ferrugineus*.

In total, 16 cultivable bacterial species were identified from the RPW larvae by 16S rRNA gene sequence analysis and were assigned into two phyla *Proteobacteria* (90.00%) and *Firmicutes*, (10.00%), belonging to four families, *Enterobacteriaceae, Streptococcaceae, Staphylococcaceae*, and *Bacillaceae* (Supplementary Table S2). Species of the genera *Enterobacter* (*E*. *cloacae* and *E*. *aerogenes*) and *Klebsiella* (*K*. *oxytoca, K*. *variicola*, and *K*. *pneumonia*) were the most dominant members, accounting for 23.00 and 20.50% in this cultivable bacteria community, respectively. The next most abundant were species of the genera *Citrobacter* (*Citrobacter freundii* and *Citrobacter koseri*) and *Cronobacter* representing 15.00 and 12.00%, respectively, of the bacterial community. The abundance of other bacterial species was less than 10.00% (**Figure 3**).

**FIGURE 4:**
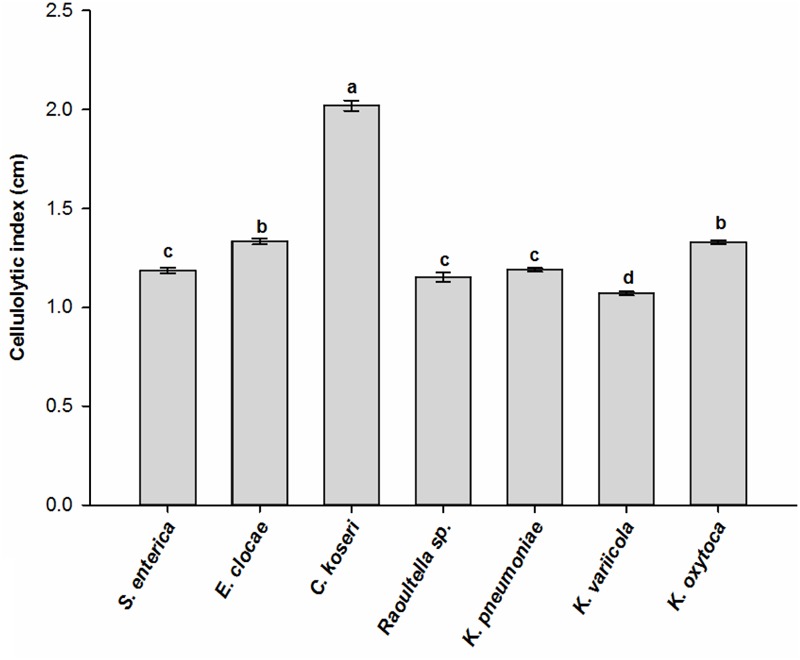
Cellulolytic activity of cultivable bacterial species isolated from the wild-caught larvae of *Rhynchophorus ferrugineus*. The different letters above the graph represent statistical significance (*p* < 0.05).

The CMC and Congo red assays revealed that seven bacterial species, *Serratia enterica, E. cloacae, Raoultella* sp., *K. pneumonia, K. variicola, K. oxytoca*, and *Citrobacter koseri*, were involved in cellulose degradation because the clearing zone was found around the colony of every bacterial species. These bacterial species performed significantly varied cellulolytic activity as they produced clear zones with different diameters ranging from 1.1 cm to 2.0 cm (ANOVA: *F*_6,14_ = 1298.04, *P* < 0.001). *Citrobacter koseri* produced the largest zones of hydrolysis, followed by *E. clocae* and *K. oxytoca*, while the species *K. variicola* showed the least hydrolytic activity (**Figure 4**). These data indicated that the gut of RPW larvae harbored bacterial species with relatively high cellulolytic activity.

### Administration of Antibiotics on the Gut Microbiota of RPW Larvae

**FIGURE 5:**
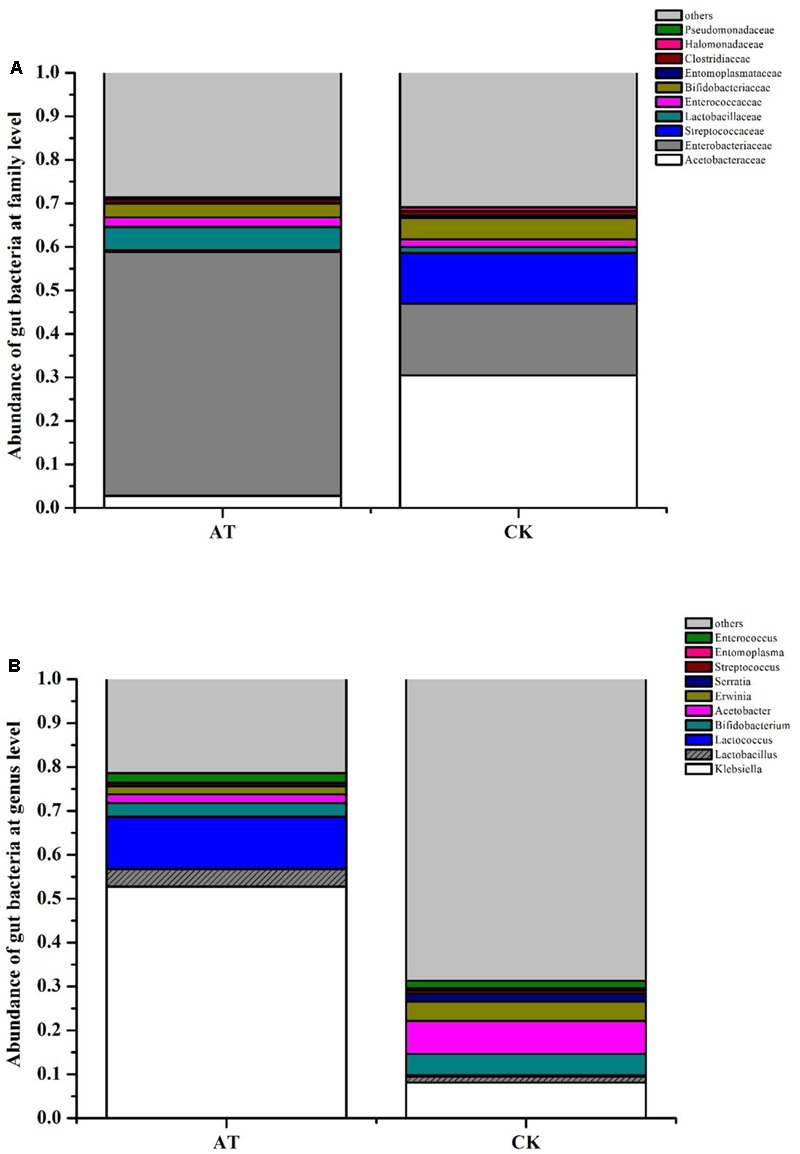
Effect of antibiotics administration on gut bacterial community composition of *Rhynchophorus ferrugineus* at family **(A)** and genus **(B)** level. AT and CK indicate the RPW larvae from antibiotics administration group and control group, respectively.

To determine the potential physiological roles of gut microbiota in RPW, antibiotics were used to disrupt the inherent homeostasis of gut microbiota in RPW larvae. After administration of antibiotics, the species richness and community diversity of larvae gut microbiota were decreased compared with CKs (**Table 2**). In the CKs, the dominant bacterial taxa were *Acetobacteraceae* and *Enterobacteriaceae* which accounted for 30.45 and 16.52%, respectively. However, their abundances were dramatically changed by antibiotics administration. For instance, the relative abundance of *Enterobacteriaceae* was increased to 56.14% (*t*-test: *t* = 4.33, *P* < 0.05) while that of *Acetobacteraceae* dropped to 2.75% (*t*-test: *t* = -10.56, *P* < 0.001, **Figure 5A**). *Klebsiella* spp. (*t*-test: *t* = 5.803, *P* < 0.05) and *Lactococcus* spp. were strongly represented in the antibiotics-feeding larvae with relative abundances of 52.77 and 11.87%, respectively (**Figure 5B**). These data indicated that gut bacterial community structure of RPW larvae was significantly altered by antibiotics administration.

**Table 2:** Richness and diversity estimation of the gut bacterial communities associated with *Rhynchophorus ferrugineus* from control (CK) and antibiotics administration (AT) groups.

Sample	Number of reads	Number of OTUs	Community diversity		Species richness	
			Shannon	Simpson	Chao1	ACE
AT1	49,226	656	3.3	0.6	704.1	713.1
AT2	58,049	679	3.6	0.7	752.8	759.7
AT3	47,644	120	2.7	0.7	145.8	149.1
CK1	51,674	794	5.8	0.9	847.0	844.6
CK2	47,260	419	4.5	0.9	480.6	488.7
CK3	48,699	637	5.9	0.9	962.7	699.9

### Perturbation of Gut Microbiota on the Nutrition Metabolism of RPW Larvae

**FIGURE 6:**
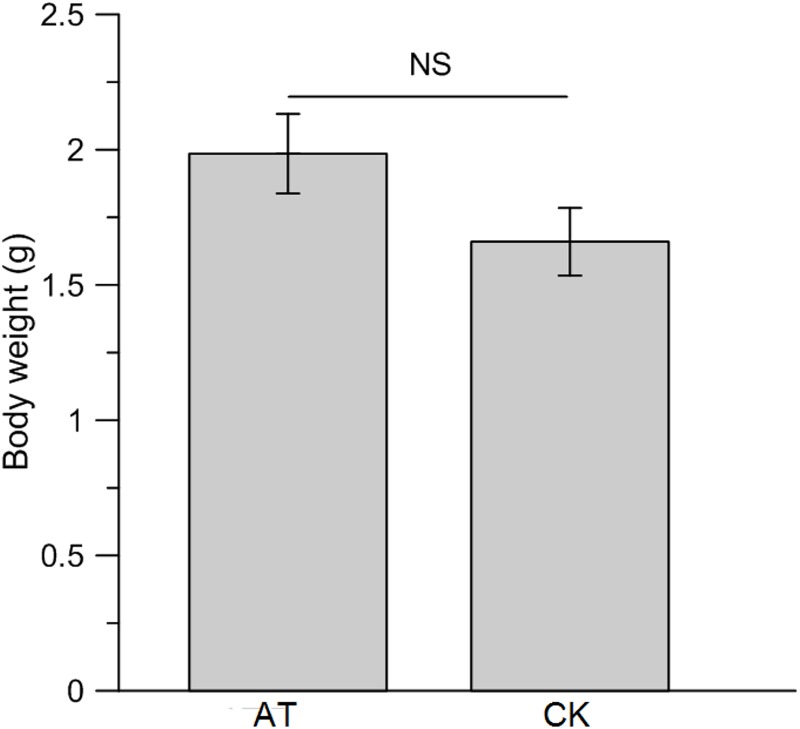
Impact of antibiotics administration-mediated alterations in gut bacterial community structure on the net body weight gain of *Rhynchophorus ferrugineus* larvae. AT and CK indicate the RPW larvae from antibiotics administration group and control group, respectively. NS, not significant.

**FIGURE 7:**
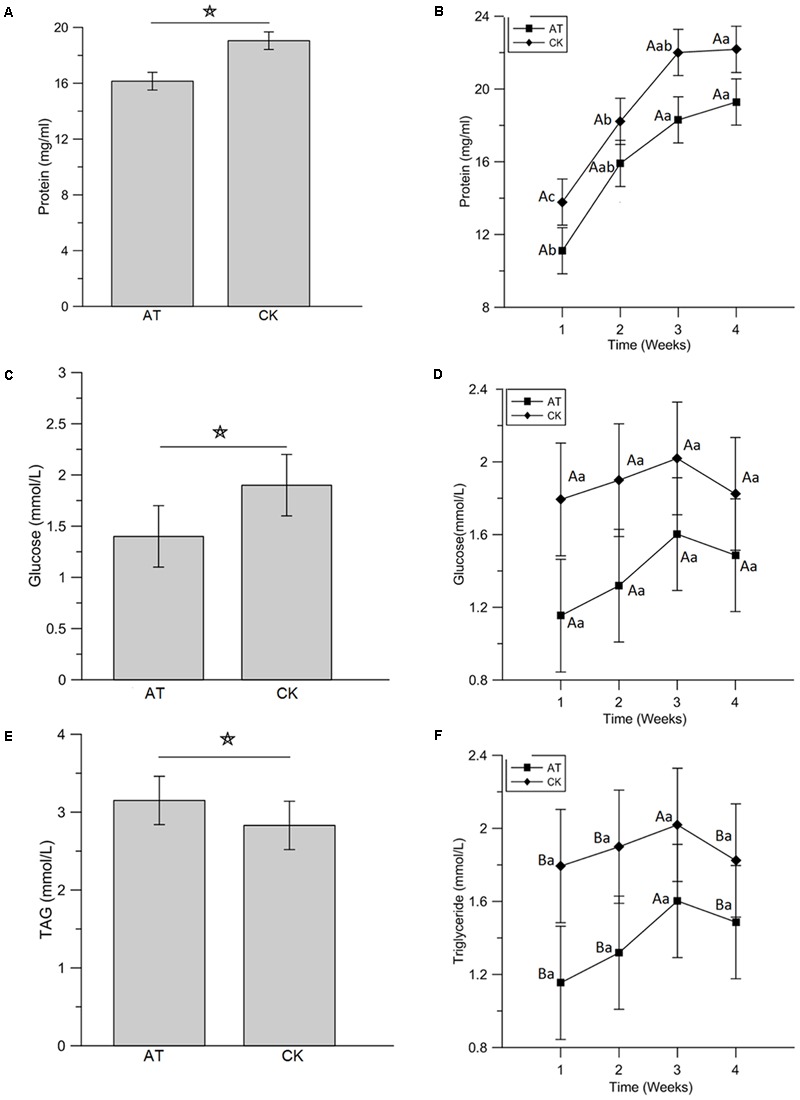
Impact of antibiotics administration-mediated alterations in gut bacterial community structure on the concentration of hemolymph protein **(A,B)**, glucose **(C,D)**, and triglyceride **(E,F)** in *Rhynchophorus ferrugineus* larvae. AT and CK indicate the RPW larvae from antibiotics administration group and control group, respectively. The star above the graphs indicates statistical significance was determined between two groups. The different uppercase and lowercase letters indicate the statistical significance was determined among different groups at the same time-point and among different time-points within the same group, respectively (*p* < 0.05).

To examine the effects of perturbation of gut microbiota on host nutrition metabolism, larva body weight, protein, glucose and TAG concentrations were measured and compared between AT and CK treatments. Although the net weight gain of antibiotics-feeding RPW larvae were higher than that of CK groups, no significant effect was determined (*t*-test: *t* = 1.68, *df* = 78, *P* > 0.05, **Figure 6**). Statistical analysis revealed that the protein level of RPW larvae was significantly reduced by the administration of antibiotics (ANOVA: *F*_1,88_ = 10.4, *P* < 0.05, **Figure 7A**). During the treatment period, the protein concentration of RPW increased with the development and the protein concentration of CK RPW were always higher than that of AT RPW (**Figure 7B**). The glucose level in AT RPW was found to be significantly lower than that in CK RPW (ANOVA: *F*_1,88_ = 5.37, *P* < 0.05, **Figure 7C**) The highest glucose level in CK and AT RPW were both recorded at the time-point of 3 weeks and then dropped 4 weeks after treatment (**Figure 7D**). By contrast, the concentration of TAG showed a distinct response to the treatments. The RPW larvae in the AT groups always contained more TAG than those in CK group during the treatment period (**Figure 7F**) and the TAG level of AT RPW was markedly higher (ANOVA: *F*_1,88_ = 12.26, *P* < 0.05, **Figure 7E**). These data highlighted that the changes in the composition of gut microbiota in RPW larvae significantly altered the host nutrition metabolism.

## Discussion

Previous surveys have found the associated gut microbiota of RPW larvae (Khiyami and Alyamani, 2008; Jia et al., 2013; Tagliavia et al., 2014) and adults (Montagna et al., 2015). However, the insect gut bacterial community is often dynamic in its quality and quantity, being determined by factors such as outside environmental habitat, diet, developmental stage and host phylogeny (Wong et al., 2011, 2015; Sudakaran et al., 2012; Blum et al., 2013; Yun et al., 2014; Montagna et al., 2015; Chen et al., 2016). Here, we disclosed that the RPW gut was mainly colonized by bacteria in the phyla *Proteobacteria* and *Firmicutes*. This result is congruent with the report on the gut metagenomes of RPW larvae from date palm (Jia et al., 2013) and lab-reared RPW adults on apple (Montagna et al., 2015). However, our RPW adults showed the second dominant taxon within Firmicutes that was scarcely found in the gut of *Phoenix canariensis*-fed RPW adults (Montagna et al., 2015). Identifying the variations of gut microbiome across various life stages and its stable indigenous gut microbiota is very important for deep elucidation of host-gut microbiota interactions. Here, we revealed that the guts of larvae, pupae and adults harbor the bacterial community primarily contained bacteria in the families *Enterobacteriaceae, Lactobacillaceae, Streptococcaceae, Enterocococcaceae*, and *Entomoplasmataceae*. *Enterobacteriaceae* being the most abundant family across the tested life stages is in line with previous studies (Jia et al., 2013; Tagliavia et al., 2014; Montagna et al., 2015). At the genus level, seven genera including *Erwinia, Entomoplasma, Lactococcus, Lactobacillus, Pseudomonas, Limnohabitans*, and *Acinetobacter* are stably represented in the gut of RPW through the whole life cycle, suggesting that they might have important effects on host fitness. Some minor differences in the abundance of major bacterial families or genera are detected with the transition of life stages in this beetle. The dynamics changes in gut microbial profiles of RPW might be caused by shifts in gut physiological conditions such as gut bacterial metabolism-mediated pH variations (Lowe et al., 1993; Engel and Moran, 2013; Jia et al., 2013). Comparisons on the diversity index revealed that RPW pupae host the highest species richness and community diversity, and gut microbiota of RPW larvae and adult are the subset of pupae’s. The higher species richness and community diversity in the pupae might be due to the fact that the gut is renewed during metamorphosis (Johnston and Rolff, 2015).

Comparative analysis on the gut bacterial consortia with RPW larvae from three species of host plants revealed that they share the almost same bacterial composition, just with changes in their abundance. For instance, although all the gut of larvae from different plant species was primarily colonized by the bacterial genera *Lactobacillus, Entomoplasma, Lactococcus, Lactococcus, Dysgonomonas*, and *Erysipelothrix*, they were detected in the RPW gut with various abundance. The stable existence of these bacterial genera implies that they may have functional importance in the gut of RPW. A higher number of OTUs were found in the larvae feeding on sugarcane in our laboratory (Supplementary Table S3), which might be caused by the reason that the dietary seeding was much more frequent, while in the field, larvae never come out from the galleries until pupation since they have little or no direct contact with the outside environment. These results suggest that RPW gut bacterial community is resilient to the changes of host plants, which is agreement with the view that diet has important roles in shaping the gut microbiota of RPW (Jia et al., 2013; Tagliavia et al., 2014; Montagna et al., 2015). Sixteen cultivable bacterial species were isolated from the gut of wild-caught RPW larvae by culture-dependent methods which helped in obtaining a better knowledge of RPW gut microbiota by cloning the full sequence of 16S rRNA gene. Interestingly, the majority of these species were members of the *Enterobacteriaceae, Streptococcaceae, Staphylococcaceae*, and *Bacillaceae* from the phyla *Proteobacteria* and *Firmicutes*, which supports the result from culture-independent methods. Collectively, the RPW gut entomotype is composed primarily of members of the *Enterobacteriaceae, Lactobacillaceae*, and *Streptococcaceae*, which may be critical for the health of RPW. Interestingly, some genus, including *Raoltella, Serritia, Enterobacter, Citrobacter, Shigell, Cronobacter, Salmonella*, and *Staphylococcus*, were not found by the pyrosequencing approach that relies upon a short 16S rRNA gene fragment. Failing of their detection could be due to the limited variability of the V4 region or their presence at very low levels.

Among the 16 species of isolated cultivable bacteria from wild-caught RPW larvae, *Enterobacter, Klebsiella, Citrobacter* and *Cronobacter* from the family *Enterobacteriaceae* are the dominant genera, representing 70.50% of this community. In the intestinal metagenomes of RPW, the open reading frames coding glycoside hydrolase have been identified in *K*. *pneumonia*, and *Citrobacter koseri* was also found being involved in the degradation of polysaccharides (Jia et al., 2013). In the present study, seven bacterial species, including *K*. *pneumonia* and *Citrobacter koseri*, were determined to have the greatest ability to degrade cellulose by in vitro CMC and Congo red assays. Isolation of cellulolytic bacteria from the gut of RPW larvae indicate that gut bacteria-mediated breakdown of plant polymers occurs inside the larvae (Butera et al., 2012). Members of the family *Enterobacteriaceae* are known as nitrogen fixers (Behar et al., 2005; Morales-Jiménez et al., 2009; Ben-Yosef et al., 2014), so the stable presence of this family in the RPW imply that gut bacteria of the *Enterobacteriaceae* can contribute to satisfy nitrogen requirements of RPW larvae feeding on nutrient-poor palm tissues, thus affecting development and fitness of RPW. Among these isolated bacteria species, *Serratia marcescens* was also found in RPW larvae gut with moderate abundance. *Serratia* plays several roles ranging from host protection against parasitoids (Costopoulos et al., 2014) to improve host fitness (Lancaster et al., 2012). The effects of *S*. *marcescens* on RPW fitness need further investigation.

The administration of antibiotics is an important alternative way to reveal the potential benefits of gut bacteria to the host by disrupting the symbiosis of gut microbiota and its host (Cho et al., 2012; Chu et al., 2013; Raymann et al., 2017). In this study, the feeding of antibiotics was found to significantly lower the community diversity and species richness of RPW larvae gut microbiota. Moreover, although no obvious variations were detected in the gut bacterial compositions, the abundance of dominant taxa was markedly changed. For example, *Enterobacteriaceae* became the first dominant taxon, followed by *Aectobacteraceae* after the administration of antibiotics. In the family *Enterobacteriaceae*, the abundance of the genus *Klebsiella* was increased significantly. In *Drosophila melanogaster* and honeybees, alteration of the gut microbiota community resulted in negative effects on host phenotype (Shin et al., 2011; Lee and Bray, 2013; Newell and Douglas, 2014; Wong et al., 2014; Clark et al., 2015; Raymann et al., 2017). Here, our evidence demonstrated that metabolic indices were dramatically affected by the antibiotics feeding-mediated alteration of gut microbiota community. Although no significance was detected in the weight gain of RPW larvae, the concentration of protein and glucose in hemolymph were reduced while that of hemolymph TAG was increased significantly by the perturbation of gut microbiota compared with CK groups. One possible reason for the reduced protein concentration might be that gut microbiota of conventional organisms improve the protein uptake by advancing the digestion of protein-rich These data highlighted that the changes in the composition of gut microbiota in RPW larvae, caused by the administration of antibiotics, significantly altered the host nutrition metabolism compounds or by synthesizing specific amino acids or by regulating the nutrient allocation (Blatch et al., 2010; Wong et al., 2014; Douglas, 2015). *Lactobacillaceae* has been uncovered to have the potential of exploiting glucose in *Drosophila* (Storelli et al., 2011; Wong et al., 2014). Interestingly, a higher abundance of *Lactobacillaceae* was detected in the AT groups with significantly lower glucose level, supporting the notion that the family *Lactobacillaceae* is involved in the metabolism of carbohydrates in RPW. Recent reports on the axenic mice (Bäckhed et al., 2004, 2007) and *Drosophila* (Ridley et al., 2012; Chaston et al., 2014) confirmed that bacteria in the family *Acetobacteraceae* are the main cause for the reduction of host TAG content. In the current investigation, the abundance of *Acetobacteraceae* obviously decreased to 2.75% which suggest that *Acetobacteraceae* function as vital partners in the lipid metabolism of RPW. Taken collectively, our evidence provided here suggests that gut bacteria function as obligate partners in the nutrition metabolism of RPW and might facilitate adaptation to exploiting new hosts in the phase of invasion. A recent report showed that collapse of gut symbiosis induced defective phenotypes in *Nezara viridula*, which present the promising notion that limitations imposed by obligate symbionts may help to counter the spread of invasive pests (Kikuchi et al., 2016; Moran, 2016). Thus, our findings provide vital implications to the development of novel pest control tactics targeting the gut bacteria of RPW. Antibiotics administartion might also have some effects on the bacteria species in hemolymph by passing through the midgut epithelial cells. This potential influence on host fitness is yet to be investigated.

## Conclusion

The composition of RPW gut microbiota is stable, being comprised of bacteria in the families *Enterobacteriaceae, Lactobacillaceae*, and *Streptococcaceae*, and the entomotype is constituted by the following most frequent genera: *Erwinia, Entomoplasma, Lactococcuss, Lactobacillus, Pseudomonas, Limnohabitans*, and *Acinetobacter*. The transition of developmental stages and host plant species show no influence on the bacterial composition, just on their abundance. Furthermore, seven species of cultivable bacteria with great cellulose degradation ability were also identified. Alterations in the gut bacteria community caused by the administration of antibiotics significantly affect the nutrition metabolism of the host, suggesting that gut bacteria are involved in RPW metabolism as the obligate partners. Our results lay strong foundations to develop novel pest management strategies for RPW by disrupting gut symbiosis to retard its further spread worldwide.

## Data Availability and Material

The Illumina high-throughput sequencing data are available in the NCBI database Sequence Read Archive (SRA) (BioProject PRJNA 389236). The Sanger sequencing data of the bacterial isolates can be accessed through accession numbers MF185369-MF185384 in the NCBI database.

## Author Contributions

ZS and YH conceived and designed the research. AM and YF performed the experiments. AM and ZS analyzed the data and wrote the manuscript. ZS and YH revised the paper and all authors approved the final version.

## Conflict of Interest Statement

The authors declare that the research was conducted in the absence of any commercial or financial relationships that could be construed as a potential conflict of interest.

## Footnotes

1 http://ccb.jhu.edu/software/FLASH/

2 http://qiime.org/index.html

3 http://drive5.com/uparse

4 http://sourceforge.net/projects/rdp-classifier/

5 http://greengenes.lbl.gov/

6 http://www.ncbi.nlm.nih.gov/blast

7 http://rdp.cme.msu.edu/seqmatch
