# Supplementary material for: The Gut Entomotype of Red Palm Weevil Rhynchophorus ferrugineus Olivier (Coleoptera: Dryophthoridae) and Their Effect on Host Nutrition Metabolism
Source: Front Microbiol. 2017 Nov 21;8:2291. doi: 10.3389/fmicb.2017.02291 (PMC5702300; doi:10.3389/fmicb.2017.02291)
Supplement: Supplementary file 1 [file Data_Sheet_1.doc]

**Supplementary Materials:**

**The Gut Entomotype of Red Palm Weevil *Rhynchophorus ferrugineus* Olivier (Coleoptera: Dryophthoridae) and Their Effect on Host Nutrition Metabolism**

**Abrar Muhammad^1, 2^, Ya Fang^1, 2^, Youming Hou^1, 2^*, Zhanghong Shi^1, 2^***

**Correspondence**

Youming Hou

[ymhou@fafu.edu.cn](mailto:ymhou@fafu.edu.cn)

Zhanghong Shi

[shizh@fafu.edu.cn](mailto:shizh@fafu.edu.cn)

**Figure legends**

**Figure S1A. Principal Component Analysis (PCA) comparing the gut microbiota between different life stages. The PC1 and PC2 are main the factors responsible for 51.62% of the variation. The Eigen values are calculated based on Bray-Curtis distance matrix for all the samples at 97% similarity threshold.**

**Figure S1B. Principal Coordinate Analysis (PCoA) comparing the gut microbiota between different life stages. The PC1 and PC2 are the factors responsible for 41.73% of the variation. The Eigen values are calculated based on Bray-Curtis distance matrix for all the samples at 97% similarity threshold.**

**Figure S2A. Principal Component Analysis (PCA) comparing the gut microbiota between different larvae groups. The PC1 and PC2 are main the factors responsible for 41.63 % of the variation.**

**Figure S2B. Principal Coordinate Analysis (PCoA) comparing the gut microbiota between different larvae groups. The PC1 and PC2 are the factors responsible for 44.06% of the variation.**


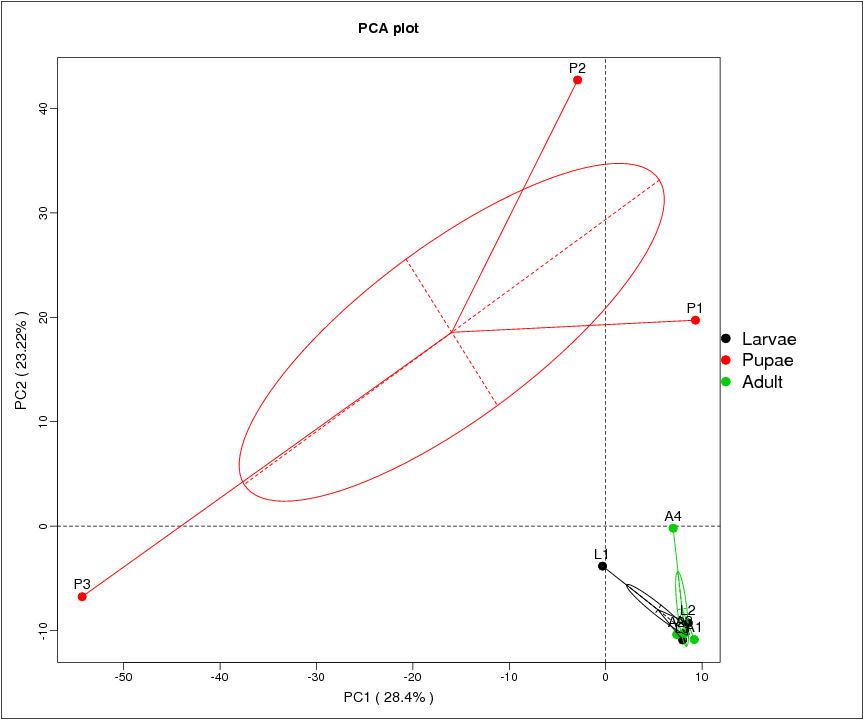


**Figure S1A**


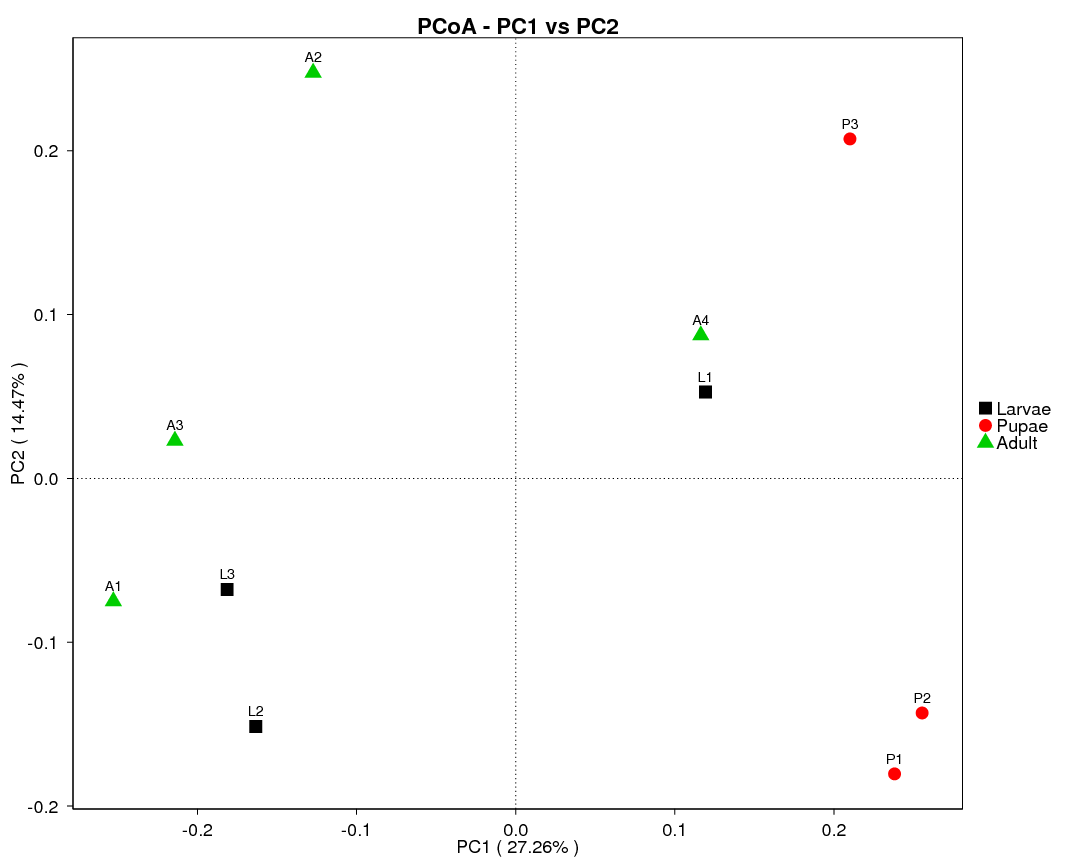


**Figure S1B**


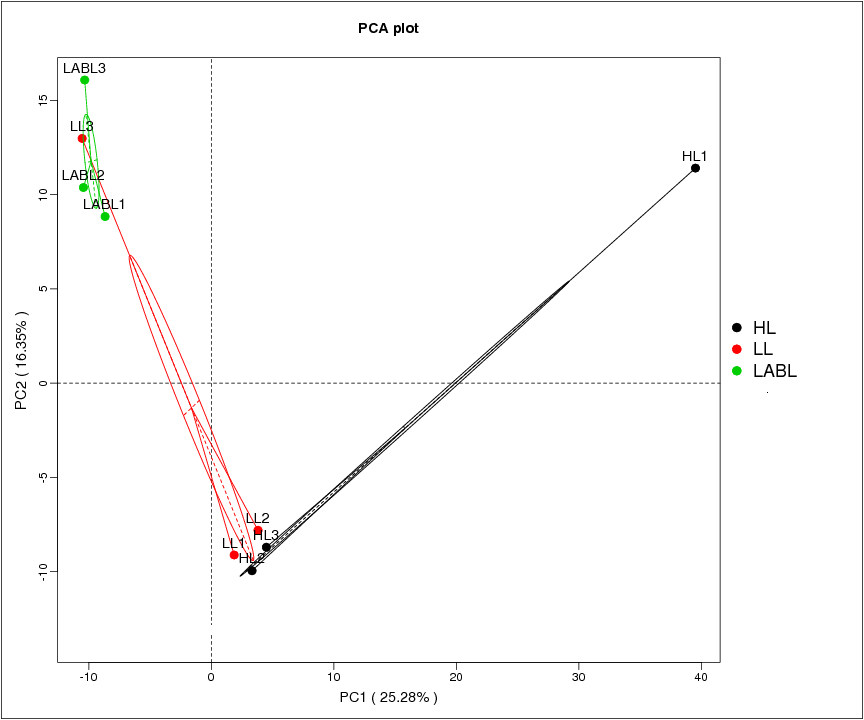


**Figure S2A**


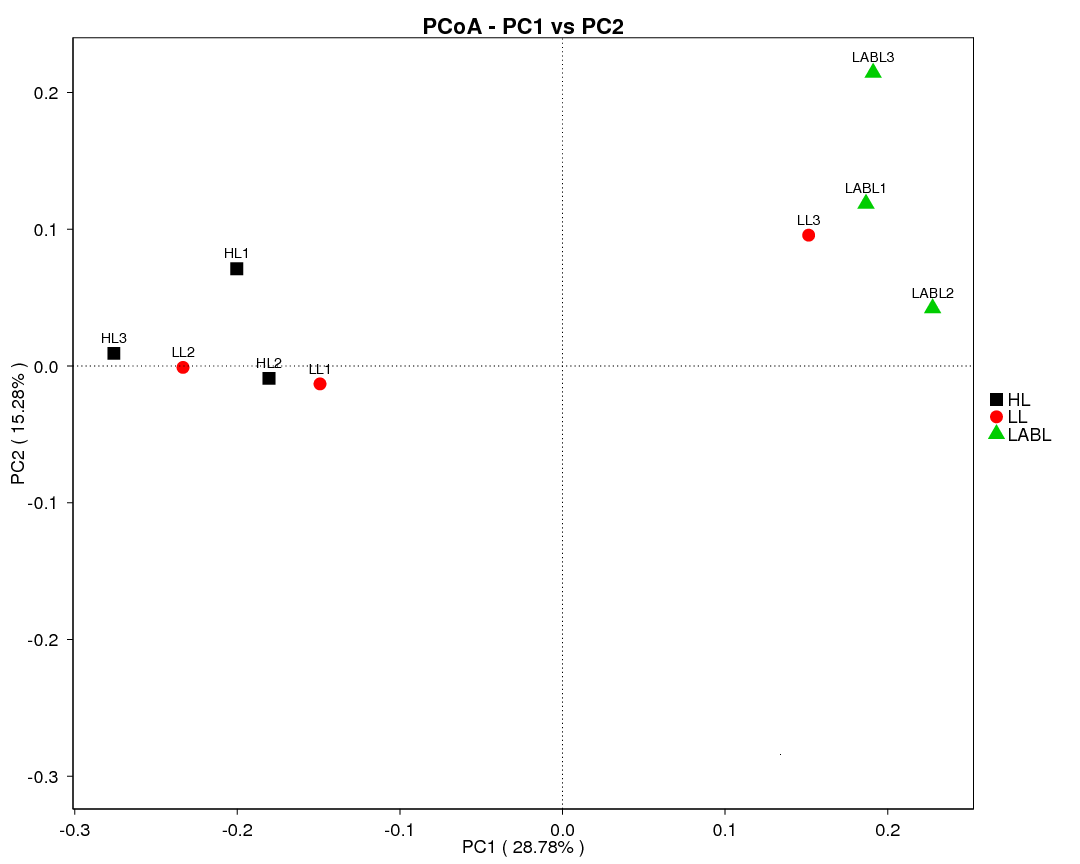


**Figure S2B**

| Table S1. Information on the specimens used in this study | | | | | |
| --- | --- | --- | --- | --- | --- |
| Samples ID | No. of  specimens | Collection locality | Latitude and Longitude | Collection date | Host plant |
| Pupae | 9 | Wenchang, Hainan | 110°72' E, 19°61' N; average elevation 42.5 m | May-2014 | *Cocos nucifera* |
| Adult | 12 | Wenchang, Hainan | 110°72' E, 19°61' N; average elevation 42.5 m | May-2014 | *Cocos nucifera* |
| Larvae HL | 9 | Wenchang, Hainan | 110°72' E, 19°61' N; average elevation 42.5 m | May-2014 | *Cocos nucifera* |
| Larvae LL | 9 | Longyan, Fujian | 117°01' E, 25°12' N; average elevation 652 m | Aug-2014 | *Phoenix canariensis* |
| Larvae LabL | 9 | FAFU, Fujian | 119°30' E, 26°08' N; average elevation 48 m | Oct-2014 | *Saccharum officinarum* |
| Larvae AT | 9 | FAFU, Fujian | 119°30' E, 26°08' N; average elevation 48 m | Jul-2015 | *Saccharum officinarum* |
| Larvae CK | 9 | FAFU, Fujian | 119°30' E, 26°08' N; average elevation 48 m | Jul-2015 | *Saccharum officinarum* |

| Table S2. Bacterial isolates identified from wild-caught *Rhynchophorus ferrugineus* larvae and their phylogenetic affiliation | | | | | | |
| --- | --- | --- | --- | --- | --- | --- |
| Strains | Closest species | Accession No. | % sequence similarity | | Family | Phylum |
| RPWL1 | *Serratia marcescens* | NR_102509.1 | | 99 | *Enterobacteriaceae* | *Proteobacteria* |
| RPWL2 | *Klebsiella oxytoca* | NR_112010.1 | | 98 | *Enterobacteriaceae* | *Proteobacteria* |
| RPWL3 | *Lactococcus lactis* | NR_113960.1 | | 99 | *Streptococcaceae* | *Firmicutes* |
| RPWL4 | *Cronobacter* sp. | NR_044059.1 | | 96 | *Enterobacteriaceae* | *Proteobacteria* |
| RPWL5 | *Raoultella* sp. | NR_044799.1 | | 98 | *Enterobacteriaceae* | *Proteobacteria* |
| RPWL6 | *Shigella* sp. | NR_026331.1 | | 99 | *Enterobacteriaceae* | *Proteobacteria* |
| RPWL7 | *Bacillus cereus* | NR_11526.1 | | 99 | *Bacillaceae* | *Firmicutes* |
| RPWL8 | *Enterobacter cloacae* | NR_117679.1 | | 98 | *Enterobacteriaceae* | *Proteobacteria* |
| RPWL9 | *Klebsiella variicola* | NR_025635.1 | | 99 | *Enterobacteriaceae* | *Proteobacteria* |
| RPWL10 | *Citrobacter koseri* | NR_104890.1 | | 99 | *Enterobacteriaceae* | *Proteobacteria* |
| RPWL11 | *Klebsiella pneumoniae* | NR_117683.1 | | 99 | *Enterobacteriaceae* | *Proteobacteria* |
| RPWL12 | *Staphylococcus saprophyticus* | NR_115607.1 | | 100 | *Staphylococcaceae* | *Firmicutes* |
| RPWL13 | *Salmonella enterica* | NR_116125.1 | | 98 | *Enterobacteriaceae* | *Proteobacteria* |
| RPWL14 | *Citrobacter freundii* | NR_028894.1 | | 99 | *Enterobacteriaceae* | *Proteobacteria* |
| RPWL15 | *Enterobacter aerogenes* | NR_102493.1 | | 99 | *Enterobacteriaceae* | *Proteobacteria* |
| RPWL16 | *Staphylococcus warneri* | NR_025922.1 | | 99 | *Staphylococcaceae* | *Firmicutes* |

Table S3. Richness and diversity estimation of the gut bacterial communities associated with *Rhynchophorus ferrugineus* larvae from different host plants from the pyrosequencing analysis

| Sample ID | Number of reads | Number of OTUs | Community diversity | | Species richness | |
| --- | --- | --- | --- | --- | --- | --- |
|  |  |  | Shannon | Simpson | Chao1 | ACE |
| HL1 | 86,024 | 492 | 3.2 | 0.7 | 1052.8 | 929.5 |
| HL2 | 99,929 | 311 | 3.6 | 0.8 | 460.8 | 545.2 |
| HL3 | 78,582 | 294 | 2.8 | 0.7 | 413.1 | 440.0 |
| LL1 | 54,385 | 283 | 2.8 | 0.7 | 355.9 | 353.1 |
| LL2 | 36,043 | 282 | 3.1 | 0.8 | 377.3 | 405.3 |
| LL3 | 41,831 | 408 | 3.7 | 0.8 | 439.5 | 472.7 |
| LABL1 | 75,912 | 415 | 3.5 | 0.8 | 432.7 | 464.6 |
| LABL2 | 56,692 | 369 | 3.3 | 0.8 | 437.2 | 465.0 |
| LABL3 | 67,403 | 402 | 3.4 | 0.8 | 389.2 | 408.9 |
